# First characterization of the complete mitochondrial genome of fungal plant-pathogen *Monilinia laxa* which represents the mobile intron rich structure

**DOI:** 10.1038/s41598-020-70611-z

**Published:** 2020-08-12

**Authors:** Gozde Yildiz, Hilal Ozkilinc

**Affiliations:** 1 Graduate School of Natural and Applied Sciences, MSc Program in Biomolecular Sciences, Canakkale Onsekiz Mart University, Çanakkale, Turkey; 2 Faculty of Arts and Sciences, Department of Molecular Biology and Genetics, Canakkale Onsekiz Mart University, Çanakkale, Turkey

**Keywords:** Computational biology and bioinformatics, Evolution, Genetics, Microbiology

## Abstract

*Monilinia laxa* is an important fungal plant pathogen causing brown rot on many stone and pome fruits worldwide. Mitochondrial genome (mitogenome) plays a critical role in evolutionary biology of the organisms. This study aimed to characterize the complete mitogenome of *M. laxa* by using next-generation sequencing and approaches of de novo assembly and annotation. The total length of the mitogenome of *M. laxa* was 178,357 bp, and its structure was circular. GC content of the mitogenome was 30.1%. Annotation of the mitogenome presented 2 ribosomal RNA (rRNA) genes, 32 transfer RNA genes (tRNA), 1 gene encoding mitochondrial ribosomal protein S3, 14 protein-coding genes and 15 open reading frame encoding hypothetical proteins. Moreover, the group I mobile introns encoding homing endonucleases including LAGLIDADG and GIY-YIG families were found both within coding regions (genic) and intergenic regions of the mitogenome, indicating an enlarged size and a dynamic structure of the mitogenome. Furthermore, a comparative mitogenomic analysis was performed between *M. laxa* and the three closely related fungal phytopathogen species (*Botryotinia fuckeliana, Sclerotinia sclerotiorum* and, *S. borealis*). Due to the number and distribution of introns, the large extent of structural rearrangements and diverse mitogenome sizes were detected among the species investigated. *Monilinia laxa* presented the highest number of homing endonucleases among the fungal species considered in the analyses. This study is the first to report a detailed annotation of the mitogenome of an isolate of *M. laxa*, providing a solid basis for further investigations of mitogenome variations for the other *Monilinia* pathogens causing brown rot disease.

## Introduction

*Monilinia laxa* is a well-known plant pathogen that causes brown rot on many stone and pome fruits. The fungus has been isolated from infected parts of shoots, blossoms, branches, and twigs of stone fruit trees (peach, cherry, plum, and apricot etc.), and pome fruit trees such as apple^1^. The pathogen could be found from blossom stage to post-harvesting stage, and results serious losses in both quantity and quality of yield^2^. Recently, brown rot of peaches has been observed in Turkey, and *Monilinia* species as related to the disease were collected and characterized^3^.

Mitochondrial genome (mitogenome) harbors useful molecular information that can be used to infer evolutionary relationships among fungal pathogens within the same genus/species and among different taxa^4,5^. For example, species detection among some *Monilinia* species was performed using intron size differences within an intron of mitochondrial *cytochrome-b* gene^6^. Mitogenome sizes may also differ within and among fungal species due to introns^7–9^. For example, mitogenome length is around 30 kb for *Candida parapsilosis*^10^ and 235 kb for *Rhizoctonia solani*^11^. Mitochondrial DNA can be circular or linear and usually are characterized by AT enriched content, and their size variation is mostly due to the presence or absence of accessory genes, mobile introns, and different lengths of intergenic regions^12^. The core gene content of the mitogenomes is largely conserved, but their relative genes order is highly variable between and within the major fungal phyla^13–15^. Furthermore, many mutations in mitogenome might be related to different traits such as virulence and drug resistance^16–19^. Thus, mitogenome information is important to find out many answers in view of the evolution and adaptation of plant pathogenic fungi. Fungal mitogenomes mainly carry the genes for ribosomal subunits, transfer RNAs, cytochrome oxidase subunits, subunits of NADH dehydrogenase, some components of ATP synthase, and some ribosomal proteins^12^. Furthermore, introns encoding open reading frames have been detected in many fungal mitogenomes^20^. These introns have been categorized as the group I and group II introns encoding homing endonucleases (LAGLIDADG and GIY-YIG) and reverse transcriptase, respectively^20^. The presence, size, number, distribution, and types of introns highly variable among the fungal species^21^. The origin, as well as the gain and the loss of introns is poorly understood^22^.

This study aimed to (i) sequence and characterize the complete mitogenome (of *M. laxa*, (ii) to determine intron types and distributions, and (iii) to compare mitogenomes of *M. laxa* and closely related species *Botryotinia fuckeliana* teleomorph of *Botrytis cinerea*), *Sclerotinia sclerotiorum* and, *S. borealis* to understand variations and dynamic structures of mitogenomes.

## Results

### General features and gene content of the mitogenome of the brown rot fungal pathogen *Monilinia laxa*

**Figure 1:**
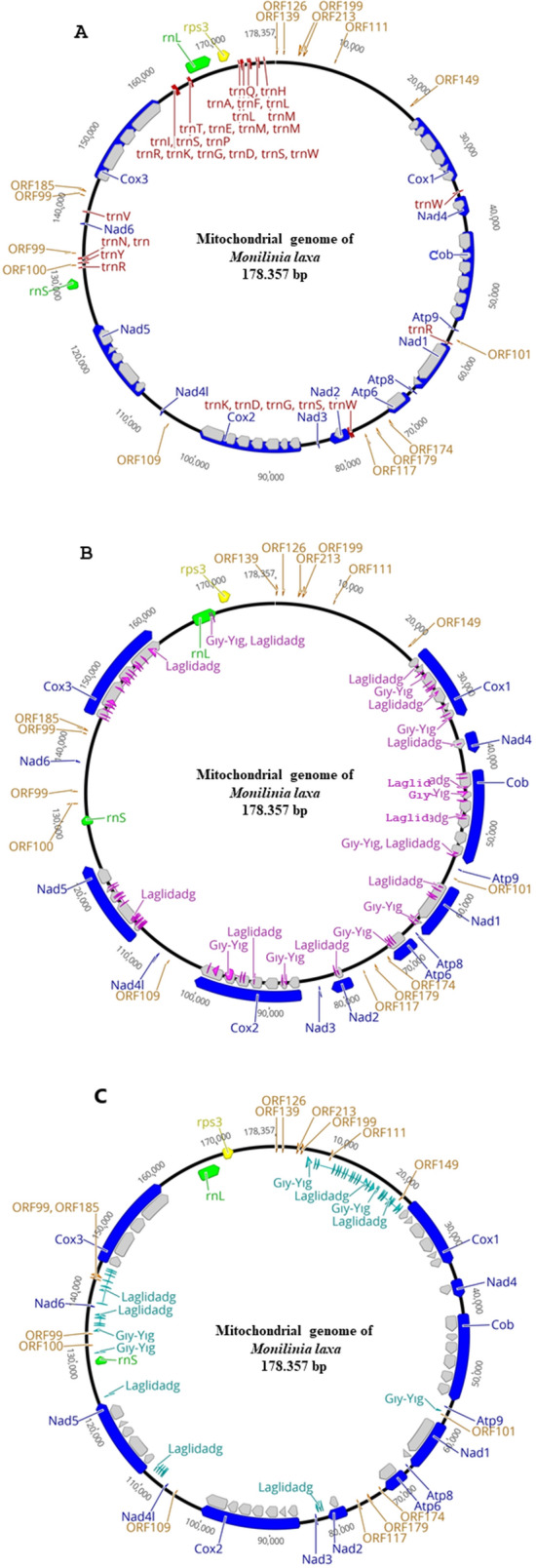
Circular map of the mitogenome of *Monilinia laxa* representing with introns (**A**) and, with group I mobile introns (HEGs) within genic regions (**B**) and intergenic regions (**C**). Genes are visualized by arrows, which are presented in a clockwise direction (forward). Blue arrows: protein-coding genes, orange arrows: hypothetical open reading frames, green arrows: genes of large and small ribosomal subunits, yellow arrow: gene encoding ribosomal protein 3, red arrows: genes of transfer RNAs, grey arrow: introns, pink arrows: HEGs within genic regions, light blue arrows: HEGs within intergenic regions. Circular mitogenomes were generated by using the Geneious 9.1.8 software^23^.

The mitogenome characterized in this study was submitted to NCBI GenBank with the accession number MN881998. The length of the mitogenome of *M. laxa* isolate Ni-B3-A2 was 178,357 bp, and included a large number of repeated sequences and many different introns (Fig. 1). The overall information about the mitogenome of *M. laxa* was as follows: T: 34.7%, C: 13.5%, A: 35.2%, and G: 16.6% and the content of GC is 30.1% with A + T-rich feature. The genome had 29 protein-coding genes (PCGs) including open reading frames for hypothetical proteins, and 14 of the coding genes were related to oxidative phosphorylation system and electron transport which were *cob*, *cox1*, *cox2*, *cox3*, *nad1*, *nad2*, *nad3*, *nad4*, *nad4L*, *nad5*, *nad6*, *atp6*, *atp8*, and *atp9* (Fig. 1; Table 1). Besides, 15 open reading frames encoded hypothetical proteins which were described as *orf139, orf126, orf213, orf199, orf111, orf149, orf101, orf174, orf179, orf117, orf109, orf100, orf99, orf185*. All the coding genes and open reading frames represented once except for *orf99,* which was named twice in the genome, but for non-homologous sequences.

**Table 1:** Characteristics and organization of annotated genes in the mitogenome of *Monilinia laxa.*

Gene	Start position	Stop position	Length (nt)	Start codon	Stop codon	GC contents	Product
*ORF139*	133	552	420	ATG	TGA	41.2	Hypothetical protein
*ORF126*	1.072	1.452	381	ATG	TGA	42.0	Hypothetical protein
*ORF213*	3.274	3.915	642	ATG	TGA	40.3	Hypothetical protein
*ORF199*	3.919	4.518	600	ATG	TGA	36.0	Hypothetical protein
*ORF111*	8.460	8.795	336	ATG	TGA	36.3	Hypothetical protein
*ORF149*	20.979	21.428	450	ATG	TAA	32.2	Hypothetical protein
*Cox1*	22.721	33.703	10.983	ATG	TGA	30.0	Cytochrome c oxidase subunit 1
*trnW*	35.302	35.372	71	–	–	36.6	Transfer RNA Tryptophan
*Nad4*	36.017	39.100	3.084	ATG	TAA	29.2	NADH dehydrogenase subunit 4
*Cob*	41.321	54.828	13.508	ATG	TAA	29.4	Apocytochrome b
*Atp9*	55.889	56.113	225	ATG	TAA	36.4	ATP synthase F0 subunit c
*ORF101*	57.205	57.510	306	ATG	TAA	40.8	Hypothetical protein
*trnR*	58.050	58.120	71	–	–	33.8	Transfer RNA Arginine
*Nad1*	58.461	66.476	8.016	ATG	TGA	30.6	NADH dehydrogenase subunit 1
*Atp8*	66.887	67.033	147	ATG	TAA	23.1	ATP synthase F0 subunit b
*Atp6*	67.840	71.727	3.888	ATG	TAA	30.1	ATP synthase F0 subunit a
*ORF174*	72.099	72.623	525	ATG	TGA	34.7	Hypothetical protein
*ORF179*	74.237	74.776	540	ATG	TAG	31.9	Hypothetical protein
*ORF117*	76.077	76.430	354	ATG	TAA	41.2	Hypothetical protein
*trnK*	77.779	77.850	72	–	–	33.3	Transfer RNA lysine
*trnG*	77.895	77.965	71	–	–	47.9	Transfer RNA lycine
*trnD*	77.967	78.039	73	–	–	41.1	Transfer RNA aspartic acid
*trnS*	78.071	78.150	80	–	–	35.0	Transfer RNA serine
*trnW*	78.195	78.265	71	–	–	35.2	Transfer RNA tryptophan
*Nad2*	78.266	81.422	3.157	ATG	TAA	29.7	NADH dehydrogenase subunit 2
*Nad3*	83.038	83.184	147	ATG	TAG	29.3	NADH dehydrogenase subunit 3
*Cox2*	85.612	100.923	15.312	ATG	TGA	30.9	Cytochrome c oxidase subunit 2
*ORF109*	105.273	105.602	330	ATG	TAG	31.8	Hypothetical protein
*Nad4L*	106.974	107.399	426	ATG	TGA	28.6	NADH dehydrogenase subunit 4L
*Nad5*	111.121	123.529	12.409	ATG	TAA	29.3	NADH dehydrogenase subunit 5
*rnS*	129.116	130.651	1.536	–	–	35.4	Small subunit ribosomal RNA
*trnR*	132.010	132.081	72	–	–	44.4	Transfer RNA Arginine
*ORF100*	132.266	132.568	303	ATG	TAA	34.7	Hypothetical protein
*trnY*	132.710	132.794	85	–	–	34.1	Transfer RNA tyrosine
*trnN*	133.373	133.443	71	–	–	38.0	Transfer RNA asparagine
*trnR*	133.466	133.537	72	–	–	47.2	Transfer RNA arginine
*ORF99*	134.002	134.301	300	ATG	TAA	24.7	Hypothetical protein
*Nad6*	138.258	138.668	411	ATT	ACT	22.6	NADH dehydrogenase subunit 6
*trnV*	140.193	140.264	72	–	–	44.4	Transfer RNA valine
*ORF99*	142.383	142.682	300	ATG	TGA	27.0	Hypothetical protein
*ORF185*	142.893	143.450	558	ATG	TAG	27.6	Hypothetical protein
*Cox3*	145.315	160.019	14.705	ATG	TCG	31.0	Cytochrome c oxidase subunit 3
*trnR*	162.780	162.850	71	–	–	35.2	Transfer RNA arginine
*trnK*	162.930	163.000	71	–	–	28.2	Transfer RNA lysine
*trnG*	163.045	163.115	71	–	–	47.9	Transfer RNA glycine
*trnD*	163.117	163.189	73	–	–	41.1	Transfer RNA aspartic acid
*trnS*	163.221	163.300	80	–	–	35.0	Transfer RNA serine
*trnW*	163.345	163.415	71	–	–	35.2	Transfer RNA tryptophan
*trnI*	165.300	165.371	72	–	–	38.9	Transfer RNA isoleucine
*trnS*	165.473	165.558	86	–	–	38.4	Transfer RNA serine
*trnP*	165.578	165.650	73	–	–	46.6	Transfer RNA proline
*rnL*	165.831	169.476	3.646	–	–	32.9	Large subunit ribosomal RNA
*rps3*	170.434	172.146	1.713	–	–	28.2	Ribosomal protein
*trnT*	173.151	173.221	71	–	–	42.3	Transfer RNA threonine
*trnE*	173.381	173.453	73	–	–	42.5	Transfer RNA valine
*trnM*	173.482	173.552	71	–	–	40.8	Transfer RNA methionine
*trnM*	173.593	173.665	73	–	–	42.5	Transfer RNA methionine
*trnL*	173.689	173.770	82	–	–	41.5	Transfer RNA leucine
*trnA*	174.477	174.548	72	–	–	40.3	Transfer RNA alanine
*trnF*	174.619	174.691	73	–	–	45.2	Transfer RNA phenylalanine
*trnL*	174.855	174.939	85	–	–	34.1	Transfer RNA leucine
*trnQ*	175.753	175.826	74	–	–	45.9	Transfer RNA glutamine
*trnH*	176.045	176.117	73	–	–	41.1	Transfer RNA histidine
*trnM*	176.812	176.884	73	–	–	31.5	Transfer RNA methionine

All 29 annotated PCGs had the same direction, and their start codon was ATG (Table 1). The preferred stop codons were TGA for the 11 PCGs, TAA for the 12 PCGs, TAG for the 4 PCGs, and TCG for 1 PCG (Table 1). Only *nad6* started with the translation initiation codon ATT and stopped with the codon ACT (Table 1). This gene also contained the highest AT frequency and the lowest GC contents (22.6%) among all the PCGs (Table 1).

Most of the genes were interrupted by introns (which are non-coding ones), as shown in Fig. 1 and Table 2. The 33 introns identified were in the core mitochondrial PCGs (Table 2). Seven introns were found in the *cox2* gene, accounting for 65.4% of the total length of the gene, which was the most intron rich gene. Intron content changed among the genes (Table 2). However, *nad3*, *nad4L*, *nad6*, *atp8-9* genes contained no intron (Table 2).

**Table 2:** Percent of intronic sequences (non-coding) present in the 14 protein coding genes of the mitogenome of *Monilinia laxa.*

Gene	No of introns	Gene size, bp	Intron size, bp	CDS size, bp^a^	% of intronic sequences
*Cox1*	6	10,983	10,338	3,756	59.9
*Cox2*	7	15,312	14,580	4,566	65.4
*Cox3*	4	14,705	14,162	6,384	52.9
*Cob*	6	13,508	12,332	3,945	62.1
*Nad1*	2	8,016	7,296	2,070	65.2
*Nad2*	1	3,157	1,459	696	24.2
*Nad3*	0	147	–	147	0
*Nad4*	1	3,084	1614	207	45.6
*Nad4l*	0	426	–	426	0
*Nad5*	5	12,409	10,411	3,492	55.8
*Nad6*	0	411	–	411	0
*Atp6*	1	3,888	3,108	1,179	49.6
*Atp8*	0	147	–	147	0
*Atp9*	0	225	–	225	0

^a^CDS size is length (nt) of introns encoded ORFs like GIY-YIG and LAGLIDADG homing endonucleases and exons of PCGs (protein coding genes).

Genes of the small and large ribosomal RNA (rRNA) subunits (*rns* and *rnl*, respectively) were identified. The sequence region of *rnl* was also invaded by introns encoding homing endonucleases (Fig. 1). Moreover, one ribosomal protein-coding gene (*rps3*) was determined.

Genome structure and order of the genes were presented in Fig. 1. All the genes identified and some information about their positions, products, lengths, GC contents, start and stop codons were described in Table 1.

### Mobile introns in the mitogenome of *Monilinia laxa*

A total of one hundred and nineteen different mobile introns were annotated in the mitogenome of *M. laxa* (Table 3). All encoding introns were characterized as the group I intron type, which encodes homing endonucleases (HE) (Table 3). Among those, the eighty-nine belonged to LAGLIDADG family, and the thirty belonged to GIY-YIG family, and were distributed within genic regions as well as intergenic regions (Fig. 1). The start codons of these elements were highly variable, but stop codons were mostly TAA, TGA, TAG (Table 3). Moreover, only two representative different stop codons were identified as ACT and AGT (Table 3). All sequences were represented once, and homology was not found among the sequences within each family. Seventy of the HE genes occupied within the intragenic regions of the mitogenome (Fig. 1). Most of these introns occupied the *cox1*, *cox2*, *cox3*, *cob*, *nad1*, *nad5* genes. The longest mobile intron sequence with 1,320 bp length was identified within *cox2* gene (Table 3). On the other hand, some genes (*atp9, atp8, nad3, nad4L, nad6*) did not show a mobile intron invasion. Sequence lengths, start-stop codons and the main location in the genome were represented for the HEs in Table 3. Locations of the different families of group I introns were illustrated in Fig. 1.

**Table 3:** The distribution and main locations of the LAGLIDADG and GIY-YIG homing endonucleases in the mitogenome of *Monilinia laxa.*

Homing endonucleases family	Lenght (bp)	Start codon	Stop codon	Location
LAGLIDADG	210	TTG	TAA	rnL
GIY-YIG	153	TTG	TAA	rnL
LAGLIDADG	1,179	GTT	TGA	Cox3
LAGLIDADG	354	GTA	TAA	Cox3
LAGLIDADG	402	ACA	TAG	Cox3
LAGLIDADG	474	ACA	TGA	Cox3
LAGLIDADG	153	AGA	TAA	Cox3
LAGLIDADG	429	AAA	TAA	Cox3
LAGLIDADG	804	GTT	TAG	Cox3
LAGLIDADG	111	GTG	TGA	Cox3
LAGLIDADG	450	GAA	TGA	Cox3
LAGLIDADG	765	TTA	TAA	Cox3
LAGLIDADG	525	AAT	TGA	Cox3
LAGLIDADG	156	ATA	TAA	Cox3
LAGLIDADG	318	AAT	TAA	Cox3
LAGLIDADG	264	ATA	TAA	Cox3
LAGLIDADG	240	TTG	TAG	Intergenic region
LAGLIDADG	291	CTT	TGA	Intergenic region
LAGLIDADG	192	ATG	TGA	Intergenic region
LAGLIDADG	201	ATG	TGA	Intergenic region
LAGLIDADG	429	GCG	TGA	Intergenic region
LAGLIDADG	633	CTA	TGA	Intergenic region
LAGLIDADG	306	CTT	TAA	Intergenic region
LAGLIDADG	573	TTA	TGA	Intergenic region
LAGLIDADG	438	GTA	TGA	Intergenic region
LAGLIDADG	168	AAG	TAA	Intergenic region
LAGLIDADG	201	GTT	TAA	Intergenic region
GIY-YIG	597	AAT	TAA	Intergenic region
GIY-YIG	168	TGT	TAA	Intergenic region
LAGLIDADG	363	ATT	TAG	Intergenic region
LAGLIDADG	279	TTA	TGA	Nad5
LAGLIDADG	264	TTT	TGA	Nad5
LAGLIDADG	132	TGC	TAA	Nad5
LAGLIDADG	351	TTG	TGA	Nad5
LAGLIDADG	417	AGA	TGA	Nad5
LAGLIDADG	147	ATG	TGA	Nad5
LAGLIDADG	474	TTA	TAG	Nad5
LAGLIDADG	132	TTT	TGA	Nad5
LAGLIDADG	267	AGC	TGA	Nad5
LAGLIDADG	105	TGT	TAA	Nad5
LAGLIDADG	396	ATG	TGA	Nad5
LAGLIDADG	213	CGT	TGA	Nad5
LAGLIDADG	96	AAC	TGA	Nad5
LAGLIDADG	96	TCG	TGA	Nad5
LAGLIDADG	123	AAT	TGA	Nad5
LAGLIDADG	369	GCA	TGA	Intergenic region
LAGLIDADG	195	AAC	TAA	Intergenic region
LAGLIDADG	312	GAG	TAA	Intergenic region
LAGLIDADG	228	AAA	TGA	Intergenic region
LAGLIDADG	144	ATC	TAA	Intergenic region
GIY-YIG	369	ATG	TAA	Cox2
GIY-YIG	939	ATG	TAA	Cox2
GIY-YIG	1,320	ATG	TAA	Cox2
GIY-YIG	237	TGC	TAA	Cox2
GIY-YIG	396	ATG	TGA	Cox2
LAGLIDADG	351	ATG	TGA	Cox2
GIY-YIG	336	ATG	TAA	Cox2
GIY-YIG	618	ATT	TGA	Cox2
LAGLIDADG	393	TCA	TAG	Intergenic region
LAGLIDADG	393	GTT	TGA	Intergenic region
LAGLIDADG	384	AAT	TGA	Intergenic region
LAGLIDADG	288	ATT	TGA	Nad2
LAGLIDADG	408	AAA	TGA	Nad2
GIY-YIG	483	ATG	TGA	Atp6
GIY-YIG	387	TTG	TAA	Atp6
GIY-YIG	309	ATG	TAG	Atp6
GIY-YIG	273	CGA	TAG	Nad1
GIY-YIG	528	AAT	TAA	Nad1
GIY-YIG	117	AAT	TGA	Nad1
GIY-YIG	486	TCA	TGA	Nad1
LAGLIDADG	273	GCA	TGA	Nad1
LAGLIDADG	393	GTT	TAG	Nad1
GIY-YIG	459	TTG	TGA	Intergenic region
LAGLIDADG	159	ATG	TAA	Cob
GIY-YIG	603	ATG	TAA	Cob
LAGLIDADG	315	ATG	TAG	Cob
LAGLIDADG	564	AAC	TGA	Cob
LAGLIDADG	423	ATT	TGA	Cob
GIY-YIG	909	TTG	TGA	Cob
LAGLIDADG	447	ATG	TAG	Cob
LAGLIDADG	396	ATG	TAA	Cob
LAGLIDADG	129	TTG	TGA	Cob
LAGLIDADG	207	ATG	TAA	Nad4
GIY-YIG	486	TTG	TAA	Cox1
GIY-YIG	309	ATG	TGA	Cox1
GIY-YIG	399	GTT	TGA	Cox1
LAGLIDADG	582	ATG	TGA	Cox1
GIY-YIG	192	ATG	TAA	Cox1
GIY-YIG	414	ATG	TGA	Cox1
GIY-YIG	306	ATG	TGA	Cox1
GIY-YIG	657	CTG	TAA	Cox1
LAGLIDADG	132	TTG	TAG	Cox1
LAGLIDADG	279	ATG	TAA	Cox1
LAGLIDADG	276	GCA	TGA	Intergenic region
LAGLIDADG	591	CCA	TGA	Intergenic region
LAGLIDADG	105	TTG	TGA	Intergenic region
LAGLIDADG	168	TCA	TGA	Intergenic region
LAGLIDADG	609	AAT	TGA	Intergenic region
LAGLIDADG	180	ATA	TGA	Intergenic region
LAGLIDADG	168	ACT	TGA	Intergenic region
LAGLIDADG	276	GTA	TGA	Intergenic region
LAGLIDADG	279	ATG	ACT	Intergenic region
GIY-YIG	159	TTG	TAG	Intergenic region
GIY-YIG	312	TTG	AGT	Intergenic region
LAGLIDADG	735	AGT	TAG	Intergenic region
LAGLIDADG	219	GTG	TGA	Intergenic region
LAGLIDADG	876	GTT	TGA	Intergenic region
LAGLIDADG	399	GTA	TAA	Intergenic region
LAGLIDADG	309	GTG	TGA	Intergenic region
LAGLIDADG	267	GTT	TGA	Intergenic region
LAGLIDADG	318	CTG	TGA	Intergenic region
LAGLIDADG	339	ACT	TGA	Intergenic region
LAGLIDADG	252	AAT	TGA	Intergenic region
LAGLIDADG	375	ACT	TAA	Intergenic region
LAGLIDADG	405	ATA	TGA	Intergenic region
LAGLIDADG	378	TTT	TGA	Intergenic region
LAGLIDADG	420	AAT	TGA	Intergenic region
LAGLIDADG	366	AAG	TGA	Intergenic region
GIY-YIG	1,032	GAG	TGA	Intergenic region

### Transfer RNAs in the mitogenome of *M. laxa*

**Figure 2:**
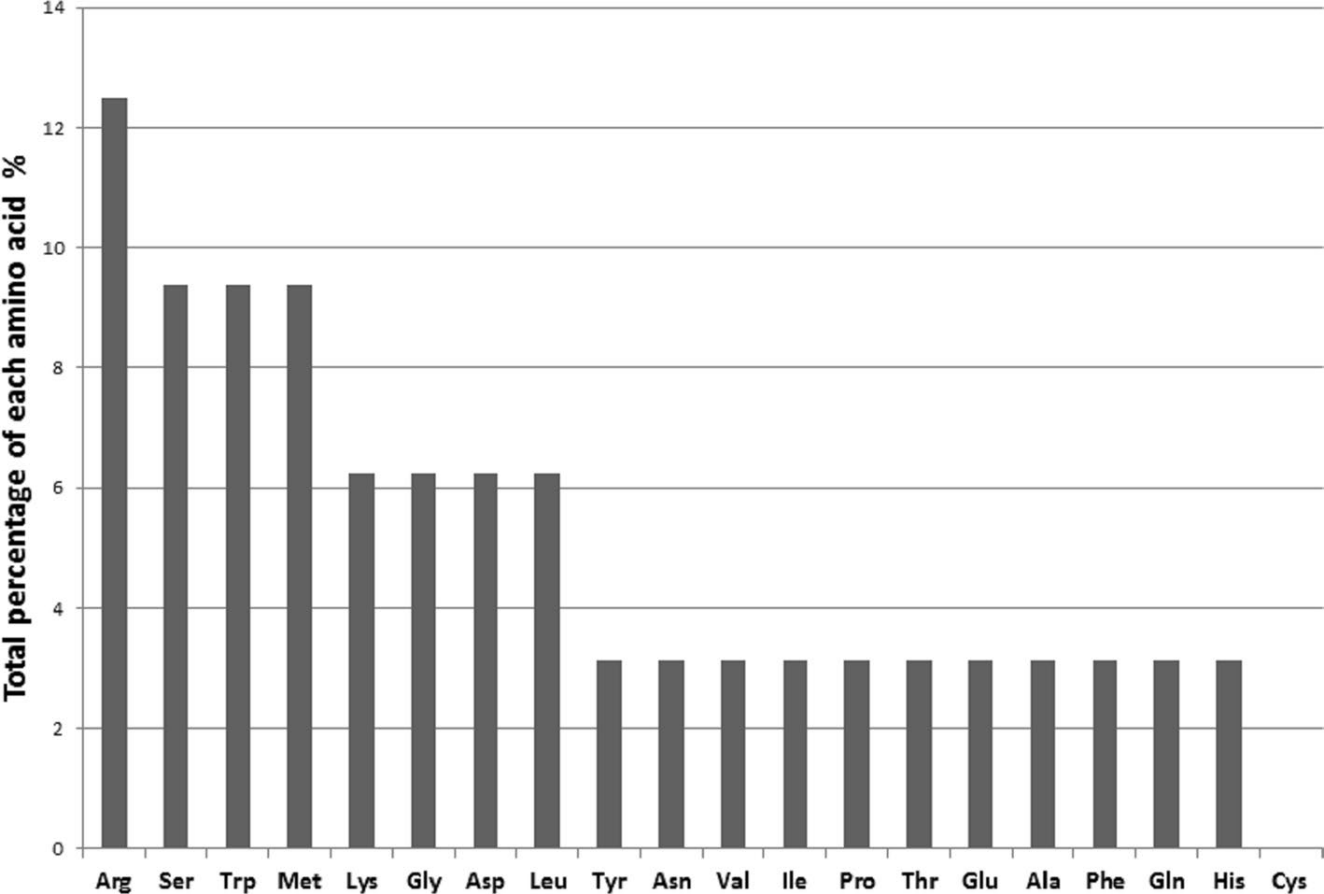
Plot depicting the percentage of amino acids carried by mitochondrial tRNAs in *Monilinia laxa* and distribution of twenty essential amino acids which are alanine (Ala), arginine (Arg), asparagine (Asn), aspartic acid (Asp), cysteine (Cys), glutamic acid (Glu), glutamine (Gln), glycine (Gly), histidine (His), isoleucine (Ile), leucine (Leu), lysine (Lys), methionine (Met), phenylalanine (Phe), proline (Pro), serine (Ser), threonine (Thr), tryptophan (Trp), tyrosine (Tyr), valine (Val). Plot was generated by using Microsoft Excel (2010).

**Figure 3:**
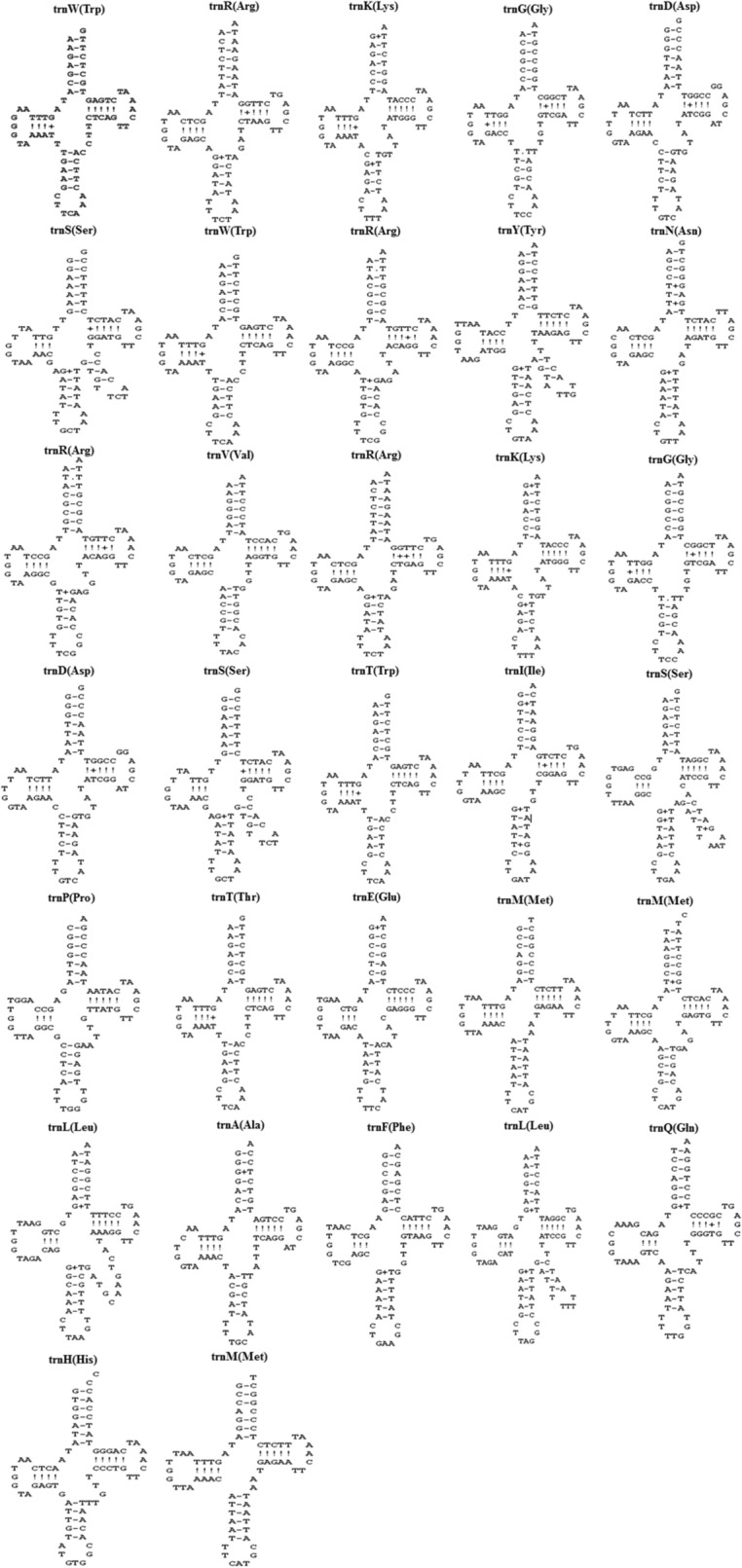
Putative secondary structures of the 32 tRNAs identified in the mitogenome of *Monilinia laxa*. The tRNAs are shown with the abbreviations of their corresponding amino acids. The map of the 32 tRNA secondary structures was drawn using the ARAGORN software^24^.

A total of 32 tRNAs associated with essential 19 amino acids were found in the mitogenome of *M. laxa* (Fig. 2). Coding for Cysteine amino acid was absent in the mitogenome of *M. laxa.* Several tRNAs were present with more than one copy: *trn-Arg* (4 copies), *trn-Ser* (3 copies), *trn-Trp* (3 copies), *trn-Met* (3 copies), *trn-Lys* (2 copies), *trn-Gly* (2 copies), *trn-Asp* (2 copies), *trn-Leu* (2) by representing different anticodon sequences (Fig. 3). Genes coding tRNAs were mostly clustered closely on the mitogenome (Fig. 1). One of the main tRNA cluster was observed in proximity of the *rnl* and *rsp3* genes, both involved in the ribosome construction process. Due to presence/absence of the extra arms, as shown in Fig. 3, tRNA sequence lengths were variable and ranged between 71 bp (*trnT*, *trnW*, *trnG*, *trnK*, *trnR*, *trnK*) and 86 bp (*trnS*).

### Comparative analyses between the mitogenomes of *M. laxa* and some closely related species

**Figure 4:**
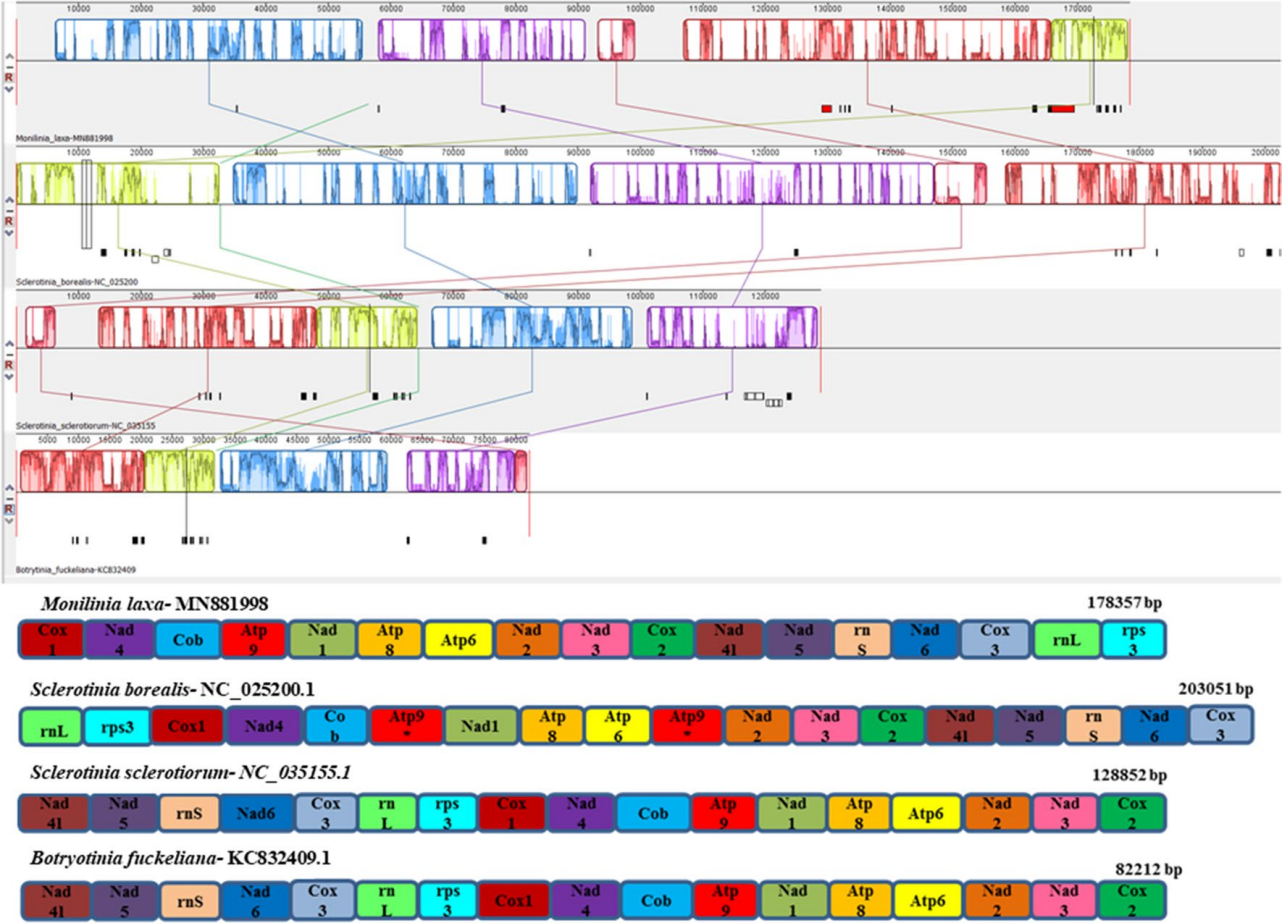
Mitogenome organizations of the four fungal species (*Sclerotinia borealis, Monilinia laxa, Sclerotinia sclerotiorum,* and *Botryotinia fuckeliana*) detected by MAUVE genome alignments, which are given different colors blocks of annotated genes. Synteny analysis was created by using MAUVE 2.3.1 software^25^.

Mitogenomes of three phytopathogenic fungi were chosen based on the results of nblast (considering the top hit values for coverage and identity) to compare the mitogenome of *M. laxa*. The selected organisms *S. borealis*, *S. sclerotiorum*, and *Botryotinia fuckeliana* belong to the same family of *M. laxa* (Table 4). The GC content was the lowest in the mitogenome of *B. fuckeliana* (29.9%) and the highest in the mitogenome of *S. borealis* (32.9%) (Table 4). All mitogenomes consisted of the core genes of mitogenomes (Table 4). The number of tRNAs varied among the species (Table 4). Genome organization was represented in Fig. 4 The conserved gene orders were the same among the species as following; *cox1, nad4, cob, atp9, nad1, atp8, atp6, nad2, nad3, cox2* (Fig. 4). Genome sizes differed among the four mitogenomes and ranged from 82 to 203 kb (Table 4). *Sclerotinia borealis* had the highest intron content, which makes the largest mitogenome size in comparison to the other species (Table 4). However, the mitogenome of *M. laxa* presented the highest content of mobile introns when compared to the other three closely related species (Table 4).

**Table 4:** Comparison of mitogenomes of *Monilinia laxa* and closely related species (*Sclerotinia borealis*, *Sclerotinia sclerotiorum* and *Botryotinia fuckeliana*).

Item	*Monilinia laxa*	*Sclerotinia borealis*	*Sclerotinia sclerotiorum*	*Botryotinia cinerea*
Division	*Ascomycota*	*Ascomycota*	*Ascomycota*	*Ascomycota*
Class	*Leotiomycetes*	*Leotiomycetes*	*Leotiomycetes*	*Leotiomycetes*
Order	*Helotiales*	*Helotiales*	*Helotiales*	*Helotiales*
Family	*Sclerotiniaceae*	*Sclerotiniaceae*	*Sclerotiniaceae*	*Sclerotiniaceae*
GenBank accession number	MN881998	NC_025200.1	NC_035155.1	KC832409.1
Genome size (nt)	178,357	203,051	128,852	82,212
GC content (%)	30.1	32.9	30.9	29.9
No. of introns	33	52	21	15
No. of standard PCGs	14	14	14	14
No. of rRNAs	2	2	2	2
No. of tRNAs	32	31	33	31
Genic regions (%)	67.1	77.38	47.49	59.28
Intergenic regions (%)	32.9	22.62	52.51	40.72
Presence of rps3	Yes	Yes	Yes	Yes
Number of GIY-YIG intragenic regions	24	22	5	7
Number of LAGLIDADG intragenic regions	46	30	6	11
Number of GIY-YIG in intergenic regions	6	2	7	1
Number of LAGLIDADG in intergenic regions	43	–	12	3
Number of Repetitive Sequences	60	62	27	20
Repetitive Sequence Motif	(AT)_17_	(T)_34_	–	(T)_35_

Intragenic (genic) regions include regions of standard PCGs (protein coding genes), open reading frame (ORFs), rRNAs, and tRNAs.

Intergenic regions include regions among standard PCGs, ORFs, rRNAs, and tRNAs.

### Repetitive sequences in the mitogenomes

Repetitive sequences detected in the mitogenomes were variable among the four species. *Sclerotinia borealis* and *M. laxa* presented the high number of repeats with total numbers 62 and 60, respectively (Table 4). Numbers of repeats were 27 and 20 in the mitogenomes of *S. sclerotiorum* and *B. fuckeliana*, respectively. The longest repeats of more than 10 bp in length were (AT)_17_ in the *M. laxa* mitogenome and this repetitive element was in an intron of *cob* gene. *Botryotinia fuckeliana* and *S. borealis* presented the same type of repetitive element, (T)_35_ and (T)_34_, respectively (Table 4). However, the location of these repetitions changed in the two species. The repetitive element located in the intergenic region between the genes *tRNA-Leu* and *tRNA-Ale* in *B. fuckeliana* while in *S. borealis* the repetitive element was found in the intergenic region between the genes *cox2* and *nad4L*. These longest repetitive sequences were found once in the mitogenomes investigated. *S. sclerotiorum* did not present any repetitive element longer than 10 bp, mostly poly A or poly T repeats inside the group I introns.

## Discussion

Currently, more than 700 complete fungal mitochondrial genomes are available, but the mitogenomes of *Monilinia* species have not been reported in the organelle genome of the NCBI database. Mitogenome of *M. fructicola* was recently announced by Ma et al.,^26^ but since this genome was not found in NCBI-blast searches, we did not use it in this study. According to the NCBI organelle genome database search, the mitogenome of *M. laxa* (isolate Ni-B3-A2) with 178,357 bp is one of the largest fungal mitochondrial genomes. Expansion of the mitogenome size has been driven by the accumulation of introns, mobile introns such as HEs, hypothetical genes, and repeats regions. Thus, increasing information about mitogenomes of plant pathogenic fungi is quite valuable. Group I introns were firstly detected in this pathogen, and these mobile elements acting as ribozyme may contribute variations within this species.

Ribosomal protein-coding genes are occasionally present in fungal mitogenomes^27–29^. *Rps3* encodes protein S3, which contributes to small ribosome assembly and this gene was identified in the isolate of *M. laxa* in this study. *Rps3* has been reported in several fungal mitogenomes, and its homolog genes have been found also in the nuclear genome for the others^28–30^. The sequence similarity and location of *rps3* are quite variable among fungal species^28,30^. In some fungal species, such as *Ophiostoma ulmi*^31^, *rnl* was detected within *rnl* group I intron^28^. The complete structure of *rps3* was not interrupted by any intron in the sequenced mitogenome of *M. laxa*. *Rps3* is highly interesting marker to evaluate evolutionary dynamics of fungal mitogenomes due to the high variability of its sequence (length, location, and rearrangement), presence, and invasion by homing endonucleases^28^.

Alternative start codon (ATT) was identified for *nad6* gene in the mitogenome of *M. laxa* in this study. These codons are suggested for mitochondrial DNA by the NCBI Genbank (https://www.ncbi.nlm.nih.gov/Taxonomy/Utils/wprintgc.cgi#SG4). Similarly, possible initiation codons were reported as TTG for *cox1* and *nad4* in the mitogenome of fungal pathogen *Scytalidium auriculariicola*^32^ and, TTG and GTG were presented as start codons for *nad2* and *cox3*, respectively, in the mitogenome of the nematode endoparasitic fungus *Hirsutella vermicola*^33^. On the other hand, some possible start codons detected in the mitogenome of fungal phytopathogen *Stemphylium lycopersici* were considered as suspicious codons and suggested that those were not acting as a start codon and the ORFs with the alternative start codons may have been co-translated with the upstream exons^34^. However, there has not been any proof of such a co-translation in the mitogenomes. Besides, different stop codons were described for fungal mitogenomes, as shown in this study and some previous studies^32–34^. Alternative transcription/translation language of mitogenomes is an interesting point of the independent evolutionary history of mitochondria and still required to be explored.

Many different HEs were discovered just in one isolate of *M. laxa*. All the sequences were non-homologous and presented different start-stop codons. Such diverse HE sequences might serve as good candidates for genome editing as reviewed by Stoddart^35^. Investigating the presence of any common HE gene in mitogenomes among the different isolates of the same species would be informative to uncover the stability of these elements at the species level. Further investigations have been performing to answer to this intriguing question.

Distribution of HEs changed among the mitochondrial genes. Moreover, the distribution of mobile introns within the same gene shows high diversity among species, as shown for *cytb* gene^36^. Mobile introns are one of the major sources for diversity and dynamics structures of mitogenomes^7,37^. Moreover, those elements may transfer horizontally among species^38^, as well as between mitogenome and nuclear genome^39^. Thus, it would be interesting to compare HEs among *Monilinia* species causing brown rot disease, and this will be pursued with our ongoing research. The mobile introns are highly interesting to understand mitogenome evolution within/among fungal species. Moreover, repetitive sequence structures varied among the species, and those elements could be used as molecular markers in population genetics and diversity analysis.

The number of tRNAs in fungal mitogenomes, even among species from the same family may vary^40^. The total tRNAs slightly differed among *M. laxa* and closely related species. Moreover, tRNAs of *M. laxa* presented different anticodons, varied lengths, and extra-arms. As an unnoticed perspective, detecting tRNAs, their structures, and related mutations within and among fungal species could be useful to investigate evolutionary changes and affected traits.

*Monilinia laxa* was compared with some closely related species. Previously, the mitogenome of *S. borealis* was compared to the known mitogenomes of helotialean fungi in a study conducted by Mardanov et al.^41^. Another research article was analyzed the mitogenomes of *Phialocephala subalpina, S. sclerotiorum* and *B. cinerea*^42^. In this study, *M. laxa* represented the highest level of intron content in comparison to the other three species. The sizes of four mitogenomes varied from 82 to 203 kb due to the different numbers of introns (Table 4). *Monilinia laxa* mitogenome (178 kb) has the second longest after *S. borealis*, while the smallest mitogenome belonged to *B. fuckeliana*. It has been observed that the genome size is directly correlated with the number and size of introns. The 14-essential protein-coding genes (*atp6, 8-9, cob, cox1-3, nad1-6,* and *nad4L*), two ribosomal RNA genes (*rnl* and *rns*) and 1 ribosomal protein coding gene (*rps3*) were observed in all these mitogenomes. However, the number of tRNAs varied between 31 and 33 across these species (Table 4). Gene orders except for anonymous ORFs and tRNAs were the same among the four mitogenomes studied.

The first mitogenome of *M. laxa* indicated a mobile intron rich structure in comparison to the closely related species, and it may differ within/between species of *Monilina* species. Our project is ongoing to obtain more mitogenome data for a large collection of *M. laxa* and *M. fructicola*.

## Materials and methods

### Fungal sample and DNA isolation

Isolates of *M. laxa* were obtained from brown-rot-diseased peach fruits in Turkey, and after pure culturing, were stored at – 20 °C on filter papers. Species identification based on both morphological criteria and polymerase chain reaction (PCR) with species-specific primers^3^. The isolate used for mitogenome characterization was obtained from the city Nigde and named Ni-B3-A2.

One piece of filter paper (approximately 0.5 mm^2^), from the long-term storage, was aseptically placed on to potato dextrose agar, incubated for one week at 23 °C in darkness. Mycelia from 7-day old culture were transferred to potato dextrose broth and incubated for 5–7 days at room temperature in a rotary shaker. Then, mycelium was harvested from the liquid using vacuum filtration. Total DNA was extracted by using Norgen Plant/Fungi DNA Isolation Kit (Norgen, Canada) following the manufacturer’s protocol. DNA quality and quantity were measured using a spectrophotometer (NanoQuant Infinite M200, Tecan) as well as fluorometer (Qubit 3.0, Thermo Fisher Scientific, USA) using the dsDNA high sensitive assay kit (Thermo Fisher Scientific, USA). Furthermore, genomic DNA was visualized on 1% agarose gel to check for any break/smear or multiple bands.

### Whole genome sequence analyses

Sequencing libraries were constituted using Illumina platform with TruSeq Nano kit to acquire as paired-end 2 × 151-bps, with about a 350-bp insert size. The next-generation sequence was performed by an external service (Macrogen Inc., Next-Generation Sequencing Service, Geumcheon-gu, Seoul, South Korea) that provided the raw sequence data. By using Trimmomatic v.36 software^43^, adapters were removed from raw reads and low-quality reads were trimmed by the setting of the parameters as LEADING and TRAINING = 10 (If their quality score is below 10, cut the bases off the start of the reads), SLIDING WINDOW = 5:20 (look at starting at base 1 and a window of 5bps, if the average quality score drops before 20, truncate the read at that position), MINLEN = 151 removing the reads shorter than 151 bps. Reads were analyzed for quality using FastQC^44^. After confirming the quality control of the sequence, data were used for further analysis.

### De novo assembly and circularization of the mitogenome of *M. laxa*

The mitogenome was extracted and assembled de novo from the whole genome data set using GetOrganelle v1.6.2^45^, which uses the implemented SPAdes v3.6.2 assembly program^46^. The best results were obtained by K-mer = 105, and mitogenome was represented as one contig. The mitochondrial genetic map was created with the Geneious 9.1.8^23^ and modified manually to circularize annotated mitogenome.

### Annotation of the mitogenome of *M. laxa*

Coding genes, introns, novel ORFs, rRNAs, and tRNAs were identified by using the online server MFannot^47^ as well as Mitos WebServer^48^. The ribosomal RNA (rRNA) subunit genes were checked by using RNAweasel^49^. The transfer RNA (tRNA) annotations were confirmed by using tRNAscan-SE 2.0^50^, and secondary structures of the tRNAs were predicted using ARAGORN^24^. Genetic Code for tRNA Isotype Prediction was used as Mold/Protozoan/Coelenterate mitochondrial genetic code. All possible open reading frames within and between genic regions were searched by using ORFinder and then checked by smart-blast of NCBI for mobile introns encoding genes.

### Comparative mitogenomics between *M. laxa *and closely related fungal species

Mitogenome of *M. laxa* was blasted using the NCBI BLAST-n tool to find the highest match with the other mitogenomes, and the highest hits were documented for the three fungal species. Thus, the mitogenomes of *Botryotinia fuckeliana* (GenBank accession number KC832409.1), *Sclerotinia sclerotiorum* (GenBank accession number NC_035155.1), *Sclerotinia borealis* (GenBank accession number NC_025200.1) were obtained from the NCBI Organelle Genome database to compare with the mitogenome of *M. laxa*. The mitogenome data obtained from the GenBank were re-annotated through MFannot^47^ to detect the number of introns. Annotated data of the four mitogenomes were compared in terms of genome sizes, structures, and contents. Comparative alignments of the whole mitogenomes were performed using MAUVE 2.3.1 software^25^, considering the annotated gene positions. The conserved regions of *M. laxa* mitogenomes were compared with the mitogenomes of *B. fuckeliana*, *S. sclerotiorum,* and *S. borealis*.

### Identification of repetitive elements

Repetitive sequences of the mitogenomes from *M. laxa*, *B. fuckeliana*, *S. sclerotiorum,* and *S. borealis* were identified. Tandem repeats were investigated by Tandem Repeats Finder (TRF)^51^ using an online interface (https://tandem.bu.edu/trf/trf.html).

### Ethical approval

This article does not contain any studies with human participants performed by any of the authors.

## Data Availability

The mitochondrial genome sequence data of the isolate of *M. laxa* used in this study was submitted to NCBI-GenBank with accession number MN881998. The mitogenomes of *B. fuckeliana*,* S. sclerotinia sclerotium*, and *S. borealis* were downloaded from NCBI-GenBank (Accession Numbers KC832409.1, NC_035155.1 and NC_025200.1, respectively).
